# Protective effect of S-adenosylmethionine on hepatic ischemia-reperfusion injury during hepatectomy in HCC patients with chronic HBV infection

**DOI:** 10.1186/1477-7819-12-27

**Published:** 2014-02-02

**Authors:** Guo-yan Liu, Wei Wang, Wei-dong Jia, Ge-liang Xu, Jin-liang Ma, Yong-sheng Ge, Ji-hai Yu, Qi-kai Sun, Fan-long Meng

**Affiliations:** 1 Anhui Key Laboratory of Hepatopancreatobiliary Surgery, 17 Lujiang Road, Hefei 230001, China; 2 Department of Hepatic Surgery, Anhui Provincial Hospital, Anhui Medical University, 17 Lujiang Road, Hefei 230001, China; 3 Anhui No.2 Provincial People’s Hospital, 1868 Dangshan Road, Hefei 230001, China; 4 Department of Medical Oncology, Anhui Provincial Hospital, Anhui Medical University, Hefei 230001, China

**Keywords:** Hepatectomy, S-adenosylmethionine, Pringle maneuver, Ischemia-reperfusion injury, Hepatitis B virus

## Abstract

**Background:**

Although hepatectomy is often performed with the Pringle maneuver, the problem of hepatic ischemia-reperfusion injury (HIRI) can also be serious. Thus, the present study was designed to investigate the protective effect of S-adenosylmethionine (SAMe) on HIRI, especially for patients with hepatocellular carcinoma (HCC) associated with chronic hepatitis B virus (HBV) infection and cirrhosis.

**Methods:**

Eighty-one HCC patients with chronic HBV infection, undergoing partial hepatectomy with inflow occlusion, were divided into three groups. In the pretreatment group (PR group, n = 26), patients were given SAMe two hours before surgery. In the post-treatment group (PO group, n = 25), patients were given SAMe six hours after surgery. And in the control group (control group, n = 30), patients received partial hepatectomy without any SAMe. All pre-, intra- and postoperative blood samples were collected to measure the plasma levels of transaminases, bilirubin and cytokines. The results were compared among the three groups.

**Results:**

There were no statistically significant intergroup differences observed in age, gender, hepatic inflow occlusion time and the results of liver function tests. Preoperative administration of SAMe (PR group) significantly reduced the plasma levels of alanine transaminase (ALT), aspartate transferase (AST), total bilirubin (TBIL) and direct bilirubin (DBIL) as compared to the other two groups. In the PO group, TBIL and DBIL were significantly lower than in the control group. Significant differences were also seen in IL-6 and TNF-α between the PR group and the other groups. In all groups, postoperative liver reserve function in the PR group as revealed by ICGR15 (Post ICGR15) was at its best before abdominal closure. Compared to the control group, the risk of complications and the hospital stay after surgery were significantly meliorated in the PR group. Additionally, patients with cirrhosis had a more acute rate of change in ALT and AST than non-cirrhotic patients.

**Conclusions:**

Taken together, our preliminary findings suggest that preoperative administration of SAMe is useful and safe for reducing the HIRI in partial hepatectomy, especially for HCC patients whose disease is associated with chronic HBV infection and cirrhosis.

## Background

Hepatocellular carcinoma (HCC), as a fatal disease, is the third leading cause of cancer death worldwide [1]. Its major cause is infection by hepatitis B virus (HBV), especially in the Asia-Pacific region. Currently, partial hepatectomy and orthotopic liver transplantation (OLT) have been considered as the most effectual treatment methods for HCC.

The Pringle maneuver is extensively applied as a standard procedure during liver resection to control blood loss, which can result in significant hepatic ischemia and reperfusion injury (HIRI). However, the amount of bleeding during surgery is closely related to higher postoperative complication rates [2]. Ischemia and reperfusion may induce oxidative stress and free radical formation [3], promote excessive serum liver enzyme release and further lead to severe organ dysfunction and organ failure. Due to the high rate of liver cell metabolism, it is an organ which is vulnerable to the influence of anoxia. However, the underlying mechanisms have not been fully clarified. To mitigate HIRI, several measures have been used in clinical application, such as ischemic preconditioning, ischemic postconditioning and pharmacological preconditioning.

S-adenosylmethionine (SAMe or AdoMet) is a natural substance and serves as a reduced glutathione, an endogenous methyl donor and ATP precursor in various tissues [4], and plays a crucial role in liver cells and especially for regulating the sensitivity of liver cells to various kinds of injuries. Chronic liver diseases lead to the depletion of SAMe [5,6], which can exacerbate liver injury as reported by preclinical studies [7]. Recently, our previous study demonstrated that postoperative SAMe therapy can provide a protective effect on residual liver function of HCC patients [8]. However, there is a lack of clinical trials focusing on how to reduce the HIRI to human livers using pharmacological strategies.

For many years, SAMe has been used extensively as a useful drug in the treatment of certain diseases [9,10]. The aim of the present study was to evaluate whether treatment with SAMe could alleviate HIRI in patients undergoing partial hepatectomy with the Pringle maneuver, especially for patients with HCC associated with chronic HBV infection and cirrhosis.

## Methods

### Patients

The protocol of this study had been approved by the Ethics Committee of Anhui Provincial Hospital affiliated to Anhui Medical University. The work undertaken conforms to the provisions of the Declaration of Helsinki. Written informed consent was obtained from each participant before surgery.

A controlled study was performed to evaluate the protective effect of SAMe on HIRI. Eighty-one consecutive patients undergoing partial hepatectomy with inflow occlusion in Anhui Provincial Hospital affiliated to Anhui Medical University (Hefei, China), between January 2011 and October 2012, were assessed for study eligibility. Inclusion criteria were (a) HCC patients with HBV infection requiring resection, and (b) no contraindications to partial hepatectomy. Exclusion criteria were listed as follows: (a) patients aged <18 or >80 years; (b) additional ablation therapies (cryosurgery or radiofrequency) or ablation therapy history; (c) liver transplantation and liver resection without inflow occlusion; (d) Child-Pugh score C liver function; (e) the indocyanine green clearance test (ICGR15) >30%, and (f) cardiovascular or cerebrovascular complications. Enrolled patients were randomly divided into three groups before surgery: (a) the pretreatment group (PR group, preconditioning with SAMe, n = 26), (b) the post-treatment group (PO group, postconditioning with SAMe, n = 25) and (c) the control group (control group, no SAMe treatment, n = 30).

### Therapeutic method

In order to minimize bias, the randomization sequence was generated by sealed and consecutively numbered envelopes providing concealment of random allocation. The same preoperative evaluation system was used on all patients and included transabdominal ultrasound, multislice spiral computed tomography (MSCT), CT angiography (CTA), three-dimensional reconstruction system to ascertain size and location of the lesion, blood biochemistry, coagulation function, chest X-ray, and electrocardiogram (ECG) to assess the patient's bodily functions. Liver function was evaluated by using the Child-Pugh grading and ICGR15 score.

Each patient was operated on by the same team of blinded and experienced surgeons. All the patients accepted anatomical liver resection. Intraoperative ultrasonography was used for all patients to accurately determine the extent of tumor and relationship with the surrounding blood vessels. The portal triad was clamped using a silastic catheter during liver parenchyma resection and continued until completion of the liver transection. The clamping time was longer than five minutes. Based on tumor location and liver function tests, anatomic resections were performed in selected patient’s liver using Kelly forceps [11], ultrasonic dissector (CUSA system, Sonoca 300, Soring, Quickborn, Germany) or ultracision harmonic scalpel (Gen 300 system, Smithfield, RI, USA). The ramifications of vessel and bile duct on the ruptured surface of remnant liver were sutured with polypropylene 4-0 or silk suture #1. The cross-section of the liver was carefully checked and suture ligated to complete hemostasis and prevent bile leakage after liver Resection. A drainage tube was placed close to the cut liver surface before closing the abdomen. Postoperative care was provided by the same group of surgeons and nurses. All patients received conventional postoperative parenteral nutrition, and early enteric nutrition was encouraged once bowel activity had recovered.

In the PR group, patients received SAMe (Abbott, Aprilia, Italy) 1,000 mg two hours before surgery and for five consecutive postoperative days. The drug was diluted in 250 ml of a 5% glucose solution and infused through a peripheral vein. The PO group also received SAMe for five consecutive days after surgery, the time to first dose being six hours after surgery. The control group underwent hepatectomy with inflow occlusion, but without SAMe administration. The administration schedule of SAMe was derived from Yang’s research [12], in which the pharmacokinetic properties of SAMe were described. The dosage of SAMe was selected according to our previous study [8] and clinical experiences.

### Data collection

Surgery data such as operating time, duration of intraoperative ischemia and the volume of blood loss were fully recorded. Postoperative outcomes such as duration of postoperative hospital stay and relative complications were also registered. The complications included surgical complications (subcutaneous fat liquefaction and infection, pulmonary infection, seroperitoneum) and post-hepatectomy liver failure (increased international normalized ratio (INR) and concomitant hyperbilirubinemia on or after postoperative day 5) [13]. ICGR15 was examined before surgery (Pre ICGR15) and before closing the abdomen (Post ICGR15) respectively, using an ICG clearance meter (DDG-2001; Nihon Kohden Industry. Co. Ltd, Tokyo, Japan).

Blood samples were collected before opening the abdomen (pre), just after surgery (AS), six hours after surgery (AS6), and on postoperative days 1 (Day 1), 3 (Day 3), 5 (Day 5), respectively. Samples were centrifuged immediately. Thereafter, plasma and serums were stored in polystyrene tubes at minus 80 degrees celsius until assay.

Alanine transaminase (ALT), aspartate transferase (AST), total bilirubin (TBIL), direct bilirubin (DBIL), prothrombin time (PT) and INR, as representing the degree of liver function, were assessed using an automated biochemistry analyzer. Plasma cytokine levels such as IL-6 and TNF-α) were measured by ELISA (R&D System, Minneapolis, MN, USA). All samples were tested in triplicate, according to the manufacturer's recommendations.

### Statistical analysis

All data were expressed as mean ± SD unless otherwise stated. All significance tests among the three groups were performed by the one-way analysis of variance (ANOVA) with Scheffe's multiple comparison procedure or Tamhane’s T2 test. If heterogeneity of variance occurred, the Fisher exact test was appropriate. In the subgroup of cirrhosis and non-cirrhosis patients, comparison between the two groups was performed using the Mann-Whitney *U*-test. *P*-values less than 0.05 were considered statistically significant.

## Results

One hundred and thirty patients were assessed for potential participants. Study exclusion criteria were concomitant availability in another randomized controlled trial (n = 15), disagreement (n = 15) and inclusion criteria not being fulfilled (n = 10). Furthermore, after randomization, there were four patients in the PR group and five in the PO group who had to be excluded from analysis (the Pringle maneuver not having been used). Finally, 26 patients were included in the PR group, 25 in the PO group, and 30 in the control group.

### Patient characteristics

Table 1 shows that no intergroup statistical differences were observed in age, gender, or the parameters of liver function tests (AST, ALT, TBIL and Pre ICGR15). All patients received liver resection for HCC, which was diagnosed by pathologic findings after surgery. Intraoperative parameters were listed in Table 2, such as operation time, duration of inflow occlusion, extent of resection, operative blood loss and background of liver histology, and showed no statistical differences.

**Table 1:** Patient backgrounds

	**PR group (n** **=** **26)**	**PO group (n** **=** **25)**	**Control group (n** **=** **30)**
Age (years)	54.92 ± 11.45	55.60 ± 12.61	52.57 ± 11.06
Male/female (%)	20/6	19/6	25/5
Prothrombin time (s)	12.54 ± 0.94	12.67 ± 1.07	12.40 ± 1.08
International normalized ratio (INR)	1.15 ± 0.09	1.19 ± 0.10	1.14 ± 0.07
ALT (u/L)	44.51 ± 22.63	44.09 ± 24.48	59.67 ± 43.1
AST (u/L)	39.03 ± 16.91	40.77 ± 19.19	42.10 ± 12.73
TBIL (μmol/L)	11.76 ± 4.32	13.32 ± 5.02	13.91 ± 4.14
Pre ICGR15 (%)	4.41 ± 3.05	6.40 ± 7.37	5.81 ± 3.68

**Table 2:** Intraoperative parameters

	**PR group (n = 26)**	**PO group (n = 25)**	**Control group (n = 30)**
Major/minor hepatectomy	7/19	7/18	9/21
Cirrhosis Ishak score (n) 0/1/2/3	5/6/13/2	4/5/11/5	4/5/16/5
Operation time (minutes)	151.69 ± 74.00	141.00 ± 56.01	130.83 ± 25.53
Pringle blood time (minutes)	15.00 ± 5.14	16.20 ± 5.90	15.90 ± 4.86
Operative blood loss (ml)	244.23 ± 266.58	284.80 ± 376.75	249.38 ± 198.63

Major resection: ≥ 3 segments; Minor resection: < 3 segments.

All patients were operated on for HCC, and 23 patients underwent a major hepatectomy (Table 2), of whom four underwent anatomic right hemi-hepatectomy (S-V to S-VIII), two underwent extended right hemi-hepatectomy (S-IV to S-VIII), three underwent anatomic left hemi-hepatectomy (S-I to S-IV) and 14 an atypical major resection. Intraoperative parameters were similar among the three groups (Table 2). The mean time of inflow occlusion was 15 minutes (PR group), 16.2 minutes (PO group) and 15.9 minutes (control group), whereas the operating time (from laparotomy to abdominal closure) was approximately two and a half hours.

### Changes of plasma levels

**Figure 1:**
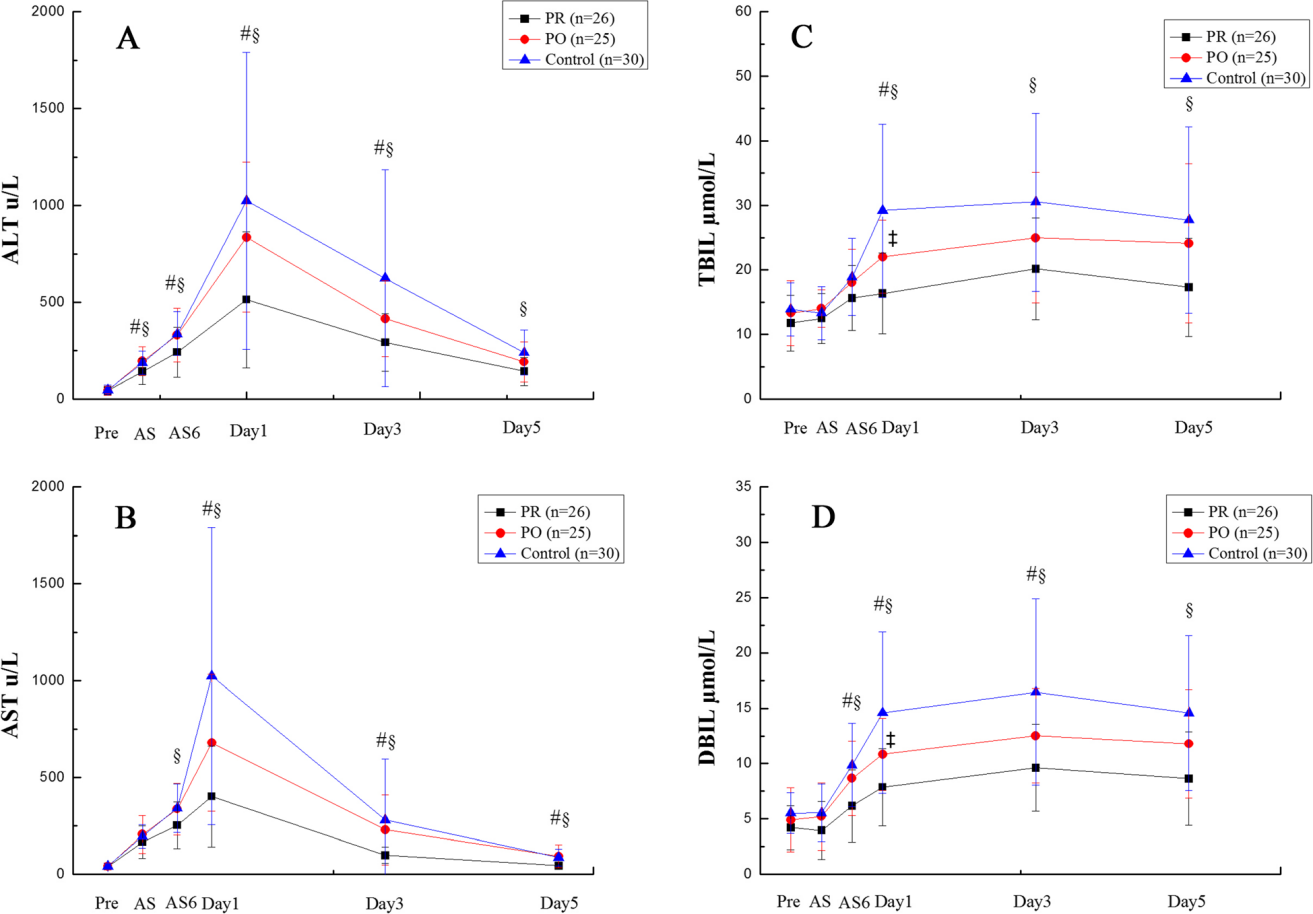
**Plasma alanine transaminase (ALT), aspartate transferase (AST), total bilirubin(TBIL) and direct bilirubin (DBIL) levels after partial hepatectomy under continuous inflow occlusion in the preconditioning group (PR), postconditioning group (PO) and without S-adenosylmethionine (SAMe) (control) group.** PR group compared with other two groups (# *P* < 0.05 versus PO group, § *P* < 0.05 versus control group), and PO group compared with control group (‡ *P* < 0.05).

Changes of plasma levels of ALT, AST, TBIL and DBIL are shown in Figure 1. Figure 1A reveals the changes in the plasma ALT at Pre, AS, AS6 and on Day1, Day3 and Day5 after surgery. At the time point of Pre, there was no significant difference among three groups. At the times AS, AS6, Day1 and Day3, the values in the PR group were significantly lower than that in PO and control groups (AS: *P* = 0.024, 0.045; AS6: *P* = 0.049, 0.023, Day1: *P* = 0.009, 0.002; Day3: *P* = 0.045, 0.011, respectively). The ALT level in the PR group was significantly lower than that in the control group (*P* = 0.003) on Day5, but there were no significant differences between the PO group and control group.

Figure 1B shows the differences in the AST level at Pre, AS, AS6 and on Day1, Day3 and Day5. At the time points of Pre and AS, there was no significant difference among the three groups. The ALT level in the PR group was significantly lower than that in the control group (*P* = 0.036) at the time point of AS6, but no significantly differences between the PO group and control group. On Day1, Day3 and Day5, the mean in PR group was significantly lower than that in the PO group and control group (Day1: *P* = 0.008, 0.001; Day3: *P* = 0.005, 0.010, Day5: *P* = 0.001, 0.000, respectively).

Figure 1C and Figure 1D show the changes in plasma TBIL and DBIL at Pre, AS, AS6 and on Day1, Day3 and Day5 after surgery. At the time points of Pre and AS, there was no significant difference among groups. Plasma TBIL was significantly lower than that in the PO and control groups on Day1 (Day1: *P* = 0.004, 0.000) and plasma DBIL was significantly lower than that in PO and control groups from AS6 to Day3 (AS6: *P* = 0.045, 0.001, Day1: *P* = 0.008, 0.000; Day3: *P* = 0.044, 0.001, respectively). The PR group was significantly lower than control group on Day3 and Day5 in TBIL (Day3: *P* = 0.003; Day5: *P* = 0.007), and for plasma DBIL, the PR group was significantly lower than the control group on Day5 (Day5: *P* = 0.001). In addition, the PO group was significantly lower than the control group on Day1 both for plasma TBIL (*P* = 0.034) and DBIL (*P* = 0.045).

### Comparison of inflammatory mediators and ICGR15

The levels of IL-6 and TNF-α were similar among the three groups before surgery. But after the administration of SAMe, IL-6 and TNF-α in the PR group (119.78 ±8.74, 228.73 ±13.42) were significantly lower than those in PO (158.62 ±17.41, 279.9 ±20.77; *P* = 0.000, 0.000) and control groups (149.95 ±13.51, 275.61 ±15.53; *P* = 0.000, 0.000), after partial hepatectomy with continuous inflow occlusion. The changes of ICGR15 before surgery (Pre ICGR15) and before closing the abdomen (Post ICGR15) also had statistical significance. The Post ICGR15 in the PR group (6.59 ±4.18) was significantly lower than that in the PO group (16.06 ±6.8, *P* = 0.000) and control group (16.14 ±8.82, *P* = 0.000).

### Comparisons of complications

In the PR, PO and control groups, complications, defined as the Clavien-Dindo classification of surgical complications [14], were listed as follows: subcutaneous fat liquefaction and infection (0, 1 (grade I) and 1 (grade I)), pulmonary infection (0, 0 and 2 (grade II)), seroperitoneum(1 (grade I), 0, and 2 (grade I)), liver failure (1 (grade I), 2 (grade I) and 5 (3 grade I, 2 grade II)), and of which 1 patient suffered from liver failure with pulmonary infection after surgery in the PR group, and 2 patients had liver failure with seroperitoneum in the control group. The postoperative complication rate (7.7%, 12.0% and 33.3%, respectively) was significantly different in the three groups (*χ*^2^ =6.410, *P* = 0.035). Also, the mean hospital stay after surgery in the PR group was significantly shorter than that in the control group (*P* = 0.044) (Table 3).

**Table 3:** Perioperative patient data

**Complications (n)**	**PR group (n** **=** **26)**	**PO group (n** **=** **25)**	**Control group (n** **=** **30)**
Complications, n (%)	2 (7.7%)	3 (12.0%)	10 (33.3%)
Hospital stay after surgery (days)	7.62 ± 1.50	9.12 ± 5.11	10.60 ± 4.49

### Comparisons of cirrhosis and non**-**cirrhosis

**Figure 2:**
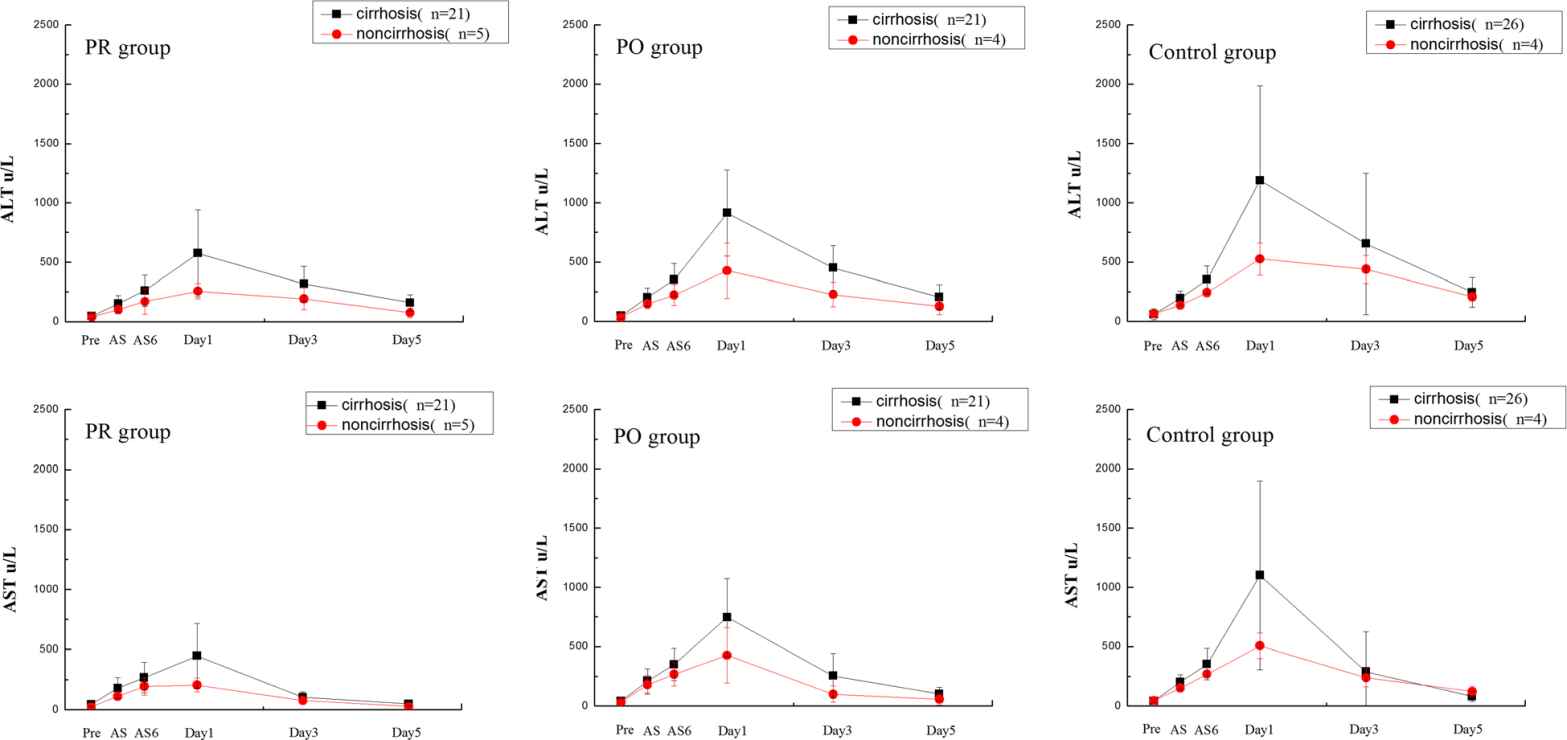
**The changes of liver function in the three groups after liver resection, analyzed in the subgroup of cirrhosis and non-cirrhosis.**

In subgroup analyses, 67 patients (82.7 %) had cirrhosis (Ishak score >1). Contrasted with non-cirrhosis patients, there were no statistical differences in preoperative data among the three groups. In the PO group, ALT, AST of non-cirrhosis patients at Day1 (*P* = 0.018, 0.032), and ALT at Day3 (*P* = 0.038) were significantly lower than in cirrhosis patients. In the PR group, ALT, AST at Day1 (*P* = 0.021, 0.015) and Day5 (*P* = 0.012, 0.007), TBIL, DBIL of non-cirrhosis patients at Day3 (*P* = 0.023, 0.016) and Day5 (*P* = 0.008, 0.008) were significantly lower than in cirrhosis patients. Moreover, the change rate between the preoperative and postoperative values in the three groups were significantly more marked in cirrhosis than that in non-cirrhosis patients (Figure 2).

## Discussion

HCC is one of the deadliest malignancies worldwide. Due to the obscure etiological agents and insufficient surveillance systems, HCC is frequently found at a late stage, often with severe liver cirrhosis and deteriorating hepatic functional reserve, especially in China. As is well known, the most important predisposing cause is infection with HBV [15]. Partial hepatectomy still remains the mainstay of therapy for HCC, not only for early stage, but also and especially for those who have large hepatic lesions. During liver surgery, clamping of the primary portahepatis is performed to control intraoperative blood loss, but this maneuver can cause serious liver function failure. Patients with cirrhosis are more impressionable to HIRI than patients with normal liver.

Under certain conditions, the protective effects of HIRI can be achieved at different time points to decrease the ischemic damage using techniques such as ischemic preconditioning [16], remote ischemic preconception [17], pharmacological preconditioning and ischemic postconditioning [18].

The present study demonstrated that the pharmacological preconditioning and postconditioning with SAMe exerted a protective effect on patients undergoing liver resection with clamping of the primary portahepatis. To the best of our knowledge, it was the first time that pharmacological preconditioning has been used in partial hepatectomy under continuous inflow occlusion, in which cirrhosis was the leading characteristic in up to 82.7% and all the patients were infected with HBV. Not only postoperative liver injury, but also the postoperative clinical outcomes were improved by pharmacological preconditioning and postconditioning. Also, the protective effect under preconditioning was much better than that under postconditioning, as represented by liver function, plasma cytokine and liver function assessment. Our preliminary results would suggest that increase of SAMe in hepatocytes through peripheral vein infusion would be advantageous in alleviating the HIRI.

SAMe is a metabolically pleiotropic molecule, which occurs in multiple cellulate reactions and serves as a key precursor of glutathione [19]. It can eliminate free radicals to deter the cellular damage [20] and act as a methyl donor in myriad biological and biochemical events [21]. Also, it participates in the reaction of transmethylation, transsulfuration and aminopropylation to preserve hepatocyte viability and proliferation. Patients with cirrhosis often have severe hepatocyte dysfunction, which can be significantly improved by increasing SAMe levels in liver cells [10,11], especially in hepatocytes after partial hepatectomy [10]. Moreover, SAMe and its metabolite can modulate proinflammatory cytokines, such as TNF-α and interleukins. Additionally, it can relieve cholestasis, which is caused by cirrhosis, viral hepatitis and surgical injury [9], the underlying mechanisms of which are frequently associated with depletion of SAMe and glutathione [20]. Recently, Liu *et al*. [22] reported that the X protein of HBV can reduce SAMe and enhance the *Mmethionine adenosyltransferase 2A* (*MAT2A*) gene expression to inhibit apoptosis in hepatocytes. However, dual effects of SAMe have also been dramatically observed. Varela-Rey *et al*. [23] found that SAMe could impair liver regeneration, and another study [24] showed it could inhibit mitogenic activity through acting on the hepatocyte growth factor (HGF). These findings were not consistent with our present study. However, due to different emphasis of study design and limitations of current reported data, the underlying mechanisms and detailed effects of SAMe need our further exploration.

The change of liver function is an extremely complex process, the details of which are difficult to elucidate. The principal biochemical parameters used as indicators of hepatocyte damage after liver resection are ALT and AST. In this study, ALT and AST in the PR group were significantly lower than those in the control group after resection, and the mean levels in the PO group after resection were lower than in the control group, but there was no statistical significance. In subgroup analyses, non-cirrhosis patients had statistically lower mean levels of ALT and AST. However, the changes in measured value were more acute in cirrhosis than in non-cirrhosis patients. These findings suggest that the cirrhotic liver was sensitive to the protective effect of SAMe.

Surgical damage during resection can lead to the elevation of serum bilirubin. Our results showed that supplying of exogenous SAMe could significantly reduce serum bilirubin in the PR group compared to that in the control group on Day1, Day3 and Day5; in the PO group, it was significantly lower than that in the control group after administration of SAMe on Day1. The underlying mechanism may be related to the fact that the supply of SAMe can restore the membrane fluidity of hepatocytes and mitochondria, strengthen the metabolism and increase the production of ATP.

ICGR15 is one of the most popular methods for examination of liver function and can reflect the operative risk before partial hepatectomy and assess the prognosis of patients with cirrhosis. Real-time ICG elimination tests [25] are often used as a new method to monitor the functional reserve of human liver. In this study, treatment with SAMe before surgery made the liver functional reserve much better than in the group without administration of SAMe.

IL-6 and TNF-α are both well-known superactive proinflammatory mediators that mediate the inflammatory response to tissue damage. Through analyzing the plasma cytokine levels, we found that preconditioning with SAMe prompted IL-6 and TNF-α production in the PR group at levels that were significantly lower than those in the PO and control groups. Considering that SAMe can modulate proinflammatory cytokines in liver injury [10], the mechanism is likely related to the downregulation of NF-κB [26]. Taking into account the change of transaminases among three groups, our data indicated that hepatocyte dysfunction after liver resection with blood inflow occlusion was mediated by the excess increase of IL-6 and TNF-α, but validation of this deduction needs further studies.

Moreover, the beneficial effects of SAMe in clinical outcomes can also be seen in this study. The patients who received SAMe had lower surgical complications, and a significantly shortened hospital stay was also observed in the PR group, mainly due to damage limitation and the rapid recovery of liver function. However, the underlying mechanisms are still unclear. Whether they work through the regulation of NF-κB, or influence the expression of some microRNAs, should be elucidated in further studies.

## Conclusions

In conclusion, our preliminary results demonstrated that preoperative administration of SAMe was useful for protecting the HIRI in HCC patients with chronic HBV infection and cirrhosis. Pharmacological preconditioning with SAMe can not only decrease liver injury after hepatic resection but also promote the life quality of patients after surgery. This strategy can be easily applied to relieve HIRI, especially in patients who have chronic HBV infection and cirrhosis.

## Consent

Written informed consent was obtained from each patient for publication of this report and any accompanying images.

## Abbreviations

ALT: alanine transaminase; ANOVA: analysis of variance; AST: aspartate transferase; CTA: CT angiography; DBIL: direct bilirubin; ECG: electrocardiogram; ELISA: enzyme-linked immunosorbent assay; HBV: hepatitis B virus; HCC: hepatocellular carcinoma; HGF: hepatocyte growth factor; HIRI: hepatic ischemia-reperfusion injury; ICGR15: indocyanine green clearance test; IL-6: interleukin-6; INR: international normalized ratio; MSCT: multislice spiral computed tomography; NF-κB: nuclear factor kappa beta; OLT: orthotopic liver transplantation; PT: prothrombin time; SAMe: S-adenosylmethionine; TBIL: total bilirubin; TNF-α: tumor necrosis factor-α.

## Competing interests

The authors declare that they have no competing interests.

## Authors’ contributions

GYL and WW are the main authors of this article as well as being responsible for data acquisition. WDJ and GYL conceived this study and WDJ led the study, participated in its design and coordination, and helped to draft the manuscript. GLX, JLM, YSG, JIY, QKS and FLM participated in study design, literature search and statistical analysis. All authors read and approved the final manuscript.
